# Nano-hydroxyapatite promotes cell apoptosis by co-activating endoplasmic reticulum stress and mitochondria damage to inhibit glioma growth

**DOI:** 10.1093/rb/rbae038

**Published:** 2024-04-18

**Authors:** Yifu Wang, Hongfeng Wu, Zhu Chen, Jun Cao, Xiangdong Zhu, Xingdong Zhang

**Affiliations:** National Engineering Research Center for Biomaterials, Sichuan University, Chengdu 610064, P. R. China; College of Biomedical Engineering, Sichuan University, Chengdu 610064, P. R. China; National Engineering Research Center for Biomaterials, Sichuan University, Chengdu 610064, P. R. China; Medical School, Kunming University of Science and Technology, Kunming 650500, P. R. China; National Engineering Research Center for Biomaterials, Sichuan University, Chengdu 610064, P. R. China; Institute of tissue engineering and stem cells, Nanchong Central Hospital, North Sichuan Medical College, Nanchong 637000, P. R. China; National Engineering Research Center for Biomaterials, Sichuan University, Chengdu 610064, P. R. China; College of Biomedical Engineering, Sichuan University, Chengdu 610064, P. R. China; National Engineering Research Center for Biomaterials, Sichuan University, Chengdu 610064, P. R. China; College of Biomedical Engineering, Sichuan University, Chengdu 610064, P. R. China; National Engineering Research Center for Biomaterials, Sichuan University, Chengdu 610064, P. R. China; College of Biomedical Engineering, Sichuan University, Chengdu 610064, P. R. China

**Keywords:** nano-hydroxyapatite, endoplasmic reticulum stress, glioma, apoptosis protein, mitochondria damage

## Abstract

Despite a growing body of studies demonstrating the specific anti-tumor effect of nano-hydroxyapatite (n-HA), the underlying mechanism remained unclear. Endoplasmic reticulum (ER) and mitochondria are two key players in intracellular Ca^2+^ homeostasis and both require Ca^2+^ to participate. Moreover, the ER–mitochondria interplay coordinates the maintenance of cellular Ca^2+^ homeostasis to prevent any negative consequences from excess of Ca^2+^, hence there needs in-depth study of n-HA effect on them. In this study, we fabricated needle-like n-HA to investigate the anti-tumor effectiveness as well as the underlying mechanisms from cellular and molecular perspectives. Data from *in vitro* experiments indicated that the growth and invasion of glioma cells were obviously reduced with the aid of n-HA. It is interesting to note that the expression of ER stress biomarkers (GRP78, p-IRE1, p-PERK, PERK, and ATF6) were all upregulated after n-HA treatment, along with the activation of the pro-apoptotic transcription factor CHOP, showing that ER stress produced by n-HA triggered cell apoptosis. Moreover, the increased expression level of intracellular reactive oxygen species and the mitochondrial membrane depolarization, as well as the downstream cell apoptotic signaling activation, further demonstrated the pro-apoptotic roles of n-HA induced Ca^2+^ overload through inducing mitochondria damage. The *in vivo* data provided additional evidence that n-HA caused ER stress and mitochondria damage in cells and effectively restrain the growth of glioma tumors. Collectively, the work showed that n-HA co-activated intracellular ER stress and mitochondria damage are critical triggers for cancer cells apoptosis, offering fresh perspectives on ER-mitochondria targeted anti-tumor therapy.

## Graphical abstract

**Figure:**
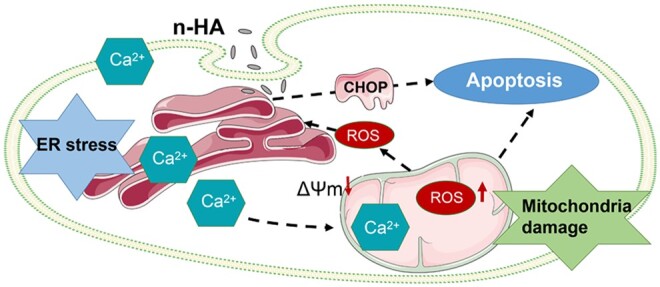

## Introduction

Among all intracranial malignancies, gliomas are the most common with a median survival time of 15 months. Despite extensive advances in the treatment of gliomas over decades, patients suffer a widespread recurrence of glioma [1, 2]. With the development of nanomaterials, researchers have reported new targets and orientation for tumor therapy, such as external stimuli-responsive systems and self-therapeutics [3, 4]. In recent decades, various unmodified or drug-free nanoparticles including nano-hydroxyapatite (n-HA) [5–8], calcium peroxide nanoparticles (CaO_2_ NPs) [9], gold nanoparticles (AuNPs) [10], nanoscopic porous amino acid mimics (nano-PAAM) [11], and others, have been reported to exhibit self-therapeutic effects on various tumors. Therefore, it can be reasonably believed that the nanoparticles with good biosafety may hold promising application potential in the treatment of glioma.

HA is a natural apatite mineral found in hard tissues, such as bones and teeth of vertebrates, and is known for its good biocompatibility, osteoconductivity, and osteoinductivity. In fact, HA existed in nature tissue is nano-scaled, and n-HA is thus synthesized and utilized to further improve biomaterials’ bioactivity [12–19]. It is interesting that some recent studies have reported the potential anti-glioma ability of n-HA [20–23]. Recently, our team has investigated the anti-tumor capabilities of n-HA in melanoma, osteosarcoma, and breast cancer [24–31], in which we discovered that needle-like n-HA exhibits superior potency in inhibiting tumor growth, along with excellent biosafety. Despite n-HA have shown significant promises in anti-tumor therapy, the underlying anti-tumor mechanism remains unclear, which limits the potential for further clinical application of n-HA.

Previous reports have shown that n-HA enters cells through clathrin-dependent endocytosis, triggers apoptosis, and ultimately limits tumor spread [24, 32]. In the tumor’s acidic microenvironment, Ca^2+^ may be liberated from n-HA during this process. Consequently, several investigations have demonstrated that n-HA causes intracellular Ca^2+^ overload, resulting in irreparable cell damage and possibly cell apoptosis [9, 33]. Generally, both the mitochondria and endoplasmic reticulum (ER) are essential for maintaining intracellular Ca^2+^ homeostasis, which also depend on Ca^2+^ to function. In addition, the interaction between the mitochondria and ER regulates the upkeep of cellular Ca^2+^ homeostasis to avert any unfavorable effects from an excess of Ca^2+^ [34]. Through the transfer of Ca^2+^ into and out of them, mitochondria and ER could modulate various cellular responses and signaling transduction pathways in response to stress, such as the prognosis and management of gliomas [35]. However, the effect of n-HA on ER and mitochondria and further cell apoptosis is rarely studied.

As reported, n-HA could activate the cell death proteases caspase-3/9 by downregulating the anti-apoptotic Bcl-2 protein in human gastric, glioma, and liver cancer cells [23, 36, 37]. Moreover, transcriptional factor C/EBP homologous protein (CHOP) was reported to trigger ER stress-mediated apoptosis by controlling Bcl-2 family members [38, 39]. In addition, mitochondrial permeability transition pore (mPTP) is a Ca^2+^-dependent channel, and the proton gradient equilibration would cause mitochondrial depolarization, further resulting in cell respiratory inhibition and reactive oxygen species (ROS) overproduction, along with mitochondria swelling and further intermembrane proteins release [40]. Furthermore, since the ER–mitochondria links serve as the physical sites for Ca^2+^, lipid, and metabolite exchange, it is expected that the Ca^2+^ and ROS signals would interact locally, and that a self-amplifying loop involving Ca^2+^-induced ROS rise and ROS-mediated Ca^2+^ vulnerability will eventually trigger cell death in concert [41, 42]. Therefore, understanding the role of the ER–mitochondria in n-HA-induced cancer cell death will be advantageous for further n-HA use and offer fresh perspectives for creating more effective ER targeted anti-tumor approaches.

Based on the above considerations, in this study, we employed the optimized needle-like n-HA to examine its anti-glioma efficacy *in vitro* and *in vivo*, meanwhile exploring the inherent mechanism from cellular and molecular perspectives. As anticipated, n-HA significantly reduced glioma cells’ ability to proliferate and invade, along with an increase in intracellular Ca^2+^ levels. It is interesting to note that the expression of ER stress indicators like phosphor-inositol requiring enzyme 1 (p-IRE1), protein kinase R-like endoplasmic reticulum kinase (PERK), and recombinant activating transcription factor 6 (ATF6) increased, and this could further lead to pro-apoptotic signaling activation via the activation of transcription factor CHOP. Additionally, increased level of intracellular ROS and the mitochondrial membrane depolarization, along with downstream apoptotic signaling pathways activation demonstrated the contribution of n-HA induced mitochondria damage to apoptosis. The *in vivo* data provided additional evidence that n-HA caused ER stress and cell apoptosis, finally resulting in glioma tumors growth inhibition. Together, the present study confirmed that intracellular ER stress and mitochondria damage, coordinately caused by n-HA, contribute to the apoptosis of glioma cells.

## Materials and methods

### Reagents

Dulbecco's modified eagle medium (DMEM) was purchased from Procell Life Science & Technology Co., Ltd (Wuhan, China). Penicillin–streptomycin and fetal bovine serum (FBS) were purchased from Shanghai BasalMedia Technologies Co., Ltd (Shanghai, China). DAB Assay Kit was obtained from ZSGB-Bio Co., Ltd (Beijing, China). Hoechst 33342/PI Assay Kit was obtained from KeyGEN Biotechnology Co., Ltd (Jiangsu, China). CCK-8 Assay Kit, EDU-594 Assay Kit, JC-1 Assay Kit and BCA Protein Assay Kit and p-IRE1α rabbit pAb were purchased from Beyotime Biotechnology Co., Ltd (Shanghai China). Anti-Cleaved Caspase-3 antibody was obtained from Abcam (Cambridge, UK). DDIT3/CHOP rabbit mAb, Caspase-3 rabbit pAb, ATF6 rabbit pAb and BiP/GRP78 rabbit mAb were purchased from ABclonal Biotechnology Co., Ltd (Wuhan, China). p-PERK pAb and PERK pAb were obtained from Thermo Fisher Scientific Co., Ltd (MA, USA).

### Characterization of n-HA

The n-HA, kindly provided by Sichuan Baiameng Bioactive Materials LLC (Chengdu, China), was characterized by several methods. The phase composition of n-HA particles was determined by X-ray diffraction (XRD, PW1050 diffractometer, Philips). Transmission electron microscope (TEM, Tecnai G2F20, USA) was used to observe the morphology and size of n-HA. Infrared spectroscopy (FTIR, Nicolet iS10 FT-IR Spectrometer, USA) was used to determine the nature of n-HA powder. The zeta potential and hydrodynamical diameter of the n-HA in DMEM were measured with a Zetasizer Nano instrument (ZS90, Malvern Instruments, Malvern, UK).

### Cell culture

Human malignant glioblastoma cells (U87MG), provided by Sebacon Biotechnology Co., Ltd (Shanghai, China), were cultured in DMEM containing penicillin–streptomycin and FBS, and then put in a standard incubator. An inverted microscope (DMI1, Leica, Germany) was performed to observe the cells morphology. Trypsin digestion was carried out, and the fresh medium was then replaced.

### Cell viability assay

After enzymatic digestion of U87MG cells, the cells were resuspended and adjusted to a density of 5 × 10^4^ cells/ml. Then, they were seeded in a 96-well plate with 100 μl per well and cultured in a standard cell incubator. After 24 h of cell culture, intervention was performed using n-HA at concentrations of 0, 200, 400, 600, 800, 1000 and 1200 μg/ml, or 400 μg/ml of NAC for 48 h. After intervention, the culture medium was removed, and 10 μl of diluted cell counting kit-8 (CCK-8) working solution was added to each well in the 96-well plate. The plate was gently swung for several times and continued to incubate. The incubation conditions were set at 37°C with 5% CO_2_ for an additional 2 h. The microplate reader was performed to measure the absorbance values for each well at 450 nm, and cell viability was calculated accordingly.

### 5-Ethynyl-2-deoxyuridine proliferation assay

U87MG glioma cells (5 × 10^4^ cells/well) were incubated in the plates and cultured for 24 h. After that, various concentrations of n-HA (0, 400, 800 and 1200 μg/ml) were added for another 48 h. A 20 μM final concentration of 5-ethynyl-2-deoxyuridine (Edu) dilution was added into the culture medium and removed following a 4 h incubation in a cell incubator at 37°C and 5% CO_2_. Subsequently, the cells were treated with 4% paraformaldehyde and incubated at 25°C for 0.5 h, then permeabilized with 0.5% Triton X-100 for 30 min at room temperature. The click additive solution was then prepared by diluting it in deionized water. Subsequently, 0.5 ml of the click reaction mixture was added to each well, and the culture plate was gently shaken and incubated in the dark for 0.5 h at 25°C. Following the removal of the click reaction mixture, Hoechst 33342 staining was performed for nuclear labeling. Finally, under fluorescence microscopy (Nikon, Japan), Edu Azide 594 exhibited red fluorescence, while Hoechst 33342 emitted blue fluorescence. Each set of experiments was repeated three times.

### Hoechst/PI double staining assay

After enzymatic digestion of U87MG cells, the cells were resuspended and adjusted to 5 × 10^4^ cells/ml. They were then seeded in a 24-well plate at 1 ml per well and cultured under conditions of 5% CO_2_ and 37°C. After the cells adhered to the surface, the intervention was performed using n-HA at concentrations of 0, 400, 800 and 1200 μg/ml. Each well had three replicates, and the experiment lasted for 48 h. At the end of the intervention period, the culture medium was removed, and Hoechst 33258 (5 μg/ml) and PI (2 μg/ml) dilution solutions were added to each well. The plate was then incubated on ice for 30 min, followed by a single wash with PBS. Subsequently, the stained cells were examined under a microscope (DMI 4000B, Leica, Germany) at emission wavelengths of 460 and 615 nm, respectively.

### Intracellular Ca^2+^ detection

Adjust the cell density to 5 × 10^4^ cells/ml at the logarithmic growth stage, seed the cell suspension into a 24-well plate, dispersing 1 ml per well, and allow the cells to culture for 24 h. Following this, add 1 ml of n-HA at different concentrations (0, 400, 800 and 1200 μg/ml) and continue cultivation for an additional 48 h. Afterwards, wash the cells twice with PBS. To determine the differences in the intracellular Ca^2+^ concentration after treatment with n-HA, glioma cells were suspended and incubated at 37°C for 45 min in darkness conditions using 5 μM Fluo-3-AM molecular probes. The cells were washed with PBS, and then 2 ml of complete culture medium was added. It was allowed to stand for 20 min. Images were captured using laser confocal microscopy (STELLARIS 5, Leica, Germany) with excitation/emission wavelengths of 506/525 nm to detect the fluorescence intensity of Ca^2+^. Repeat each experimental set three times to ensure robust data.

### Intracellular ROS assay

Adjust the cell density to 5 × 10^4^ cells/well and seed in a 24-well plate with 1 ml per well, then culture under standard conditions. Once the cells adhered to the surface, intervention was performed using n-HA at concentrations of 0, 400, 800 and 1200 μg/ml for 48 h. Subsequently, the cells were washed with PBS, then suspended and co-cultured with serum-free culture medium containing 10 μM DCFH-DA. The cultivation temperature was set at 37°C and maintained for 1 h under dark conditions. Following another wash with PBS, the cells were observed using laser confocal microscopy. Conduct each experimental set thrice for reliable and consistent data.

### Intracellular JC-1 staining

The mitochondrial membrane potential (ΔΨm) in U87MG cells was tested using the JC-1 fluorescence probe. Firstly, U87MG cells (5 × 10^4^ cells/well) were treated with different concentrations of n-HA (0, 400, 800 and 1200 μg/ml) for 48 h. Subsequently, 2 µl of JC-1 fluorescence probe was added to each well, and incubated at 37°C in darkness for 15 min. After washing twice with PBS and adding fresh culture medium, fluorescence was examined using a fluorescence microscope. The change in ΔΨm was indicated by the transition of JC-1 monomers (green, 530 nm) into aggregates (red, 590 nm), observable through the fluorescence microscope.

### Western blotting

After treating U87MG glioma cells (5 × 10^4^ cells/well) with various concentrations of n-HA (0, 400, 800 and 1200 μg/ml) for 48 h, the cells were harvested. Protein was extracted from the cells using RIPA lysis buffer supplemented with 1 mM PMSF and quantified with a BCA kit. Subsequently, the protein was separated by SDS-PAGE and transferred onto polyvinylidene difluoride (PVDF) membranes. These membranes were then blocked with 5% non-fat milk for 1 h at 25°C. After the blocking step, the membranes were incubated with primary antibody at 4°C for 12 h. The next day, after washing the membranes with TBST and TBS, they were incubated with the secondary antibody for 2 h at 25°C. The grayscale values were determined by a Bio-blot exposure meter (5200 Multi, Shanghai, China). Each set of experiments was repeated three times.

### Wound healing and transwell migration assay

The migration of glioma cells was studied through a wound healing experiment. U87MG cells, seeded at a concentration of 5 × 10^4^ cells/well in a 24-well plate, were allowed to grow until they reached 90–100% confluency. Following this, the culture medium was aspirated, and a scratch was created vertically across the well using a pipette tip. After washing three times with PBS to eliminate detached cells along the scratch, various concentrations of n-HA (0, 400, 800 and 1200 μg/ml) were introduced, and the cells were incubated for an additional 48 h. Images of the plate were captured using a microscope at 0, 24 and 48 h post-treatment with n-HA.

The transwell assay (Corning Inc., USA) was performed to evaluate the invasion ability of glioma cells. Matrigel matrix gel (BD Biosciences, USA) was pre-chilled overnight in a 4°C refrigerator. The next day, the transwell chambers were thoroughly soaked in PBS and allowed to air-dry. Subsequently, a serum-free medium was used to dilute the Matrigel gel matrix in a 1:8 ratio, and 30 μl of this diluted mixture was added to the upper chamber. After equilibrating at 4°C for 60 min, the chambers were placed in a 37°C incubator for 3 h to allow the Matrigel matrix gel to spread evenly in the upper chamber. The U87MG cell concentration was then adjusted to 2 × 10^5^ cells/200 μl. Subsequently, 200 μl of cell suspension and different concentrations of n-HA (0, 400, 800 and 1200 μg/ml) were added to the upper chamber, while 600 μl of complete culture medium was added to the lower chamber. The samples were then incubated for an additional 48 h. Following this, the culture medium was removed, excess cells were wiped away with a cotton swab, and the plates were washed with PBS. The cells were fixed with 4% paraformaldehyde and stained for 10 min with 0.4% crystal violet. Three random fields were selected for photography, and the number of cells in the lower chamber was counted. This entire process was repeated three times.

### Glioma animal model

The Ethics Committee of Animal Research at Sichuan University granted approval for the animal experimentation conducted in this study (ethics number KS2021635). All experimental procedures adhered strictly to the relevant national guidelines established by the Ministry of Science and Technology. A total of 21 BALB/c nude mice (weighing 16–18 g, sourced from Chengdu Dashuo Laboratory Animal Co., LTD., License No. SCXK [Si Chuan] 2020-030) were used to establish the *in vivo* animal model for glioma inhibition based on our previous methods [26–29]. The experimental animals were divided into three groups: a control group, a low-dose n-HA group and a high-dose n-HA group. In the control group, a mixture composed of Matrigel (100 µl) and PBS (100 µl) containing 1 × 10^7^ tumor cells was injected into the back of each mouse. In both n-HA groups, the specified doses of n-HA (50 mg/kg for the low-dose group and 100 mg/kg for the high-dose group) were added to the PBS suspension, and the mixture was immediately injected into the mice after being combined with Matrigel. Following the injection, the body weight of each mouse and tumor growth were closely monitored and recorded at designated time points.

### *In vivo* anti-glioma evaluation

The length (*L*) and width (*W*) of tumors in nude mice were measured every 3 days. Tumor volume was calculated using the formula (*L* × *W*^2^)/2. At the end of the 28-day observation period, all the animals were humanely euthanized, and the tumor tissues were collected and weighed.

### Hematoxylin–eosin staining

The excised tumor specimens were fixed in 4% paraformaldehyde and underwent paraffin embedding. After deparaffinization, the longitudinal sections were stained with hematoxylin for 5 min. Subsequently, the sections were soaked in 1% acid ethanol for 5 min and rinsed in distilled water. They were then stained with eosin for 3 min, dehydrated using a graded alcohol series, cleared in xylene and mounted. Finally, the sections were observed under an optical microscope.

### TUNEL detection

The excised tumor specimens were fixed in 4% paraformaldehyde and underwent paraffin embedding. After deparaffinization, the longitudinal sections were stained with hematoxylin for 5 min. Subsequently, the sections were soaked in 1% acid ethanol for 5 min, rinsed in distilled water, stained with eosin for 3 min, dehydrated using a graded alcohol series, cleared in xylene, and mounted. Finally, the sections were observed under an optical microscope.

### Immunohistochemistry assay

The expression of CD31, Ki67, Cleaved Caspase-3, CHOP, or p-PERK in the tissue sections was assessed using immunohistochemistry (IHC) staining. Paraffin-embedded sections were deparaffinized and underwent gradient ethanol hydration. Subsequently, antigen retrieval was performed using an antigen retrieval solution and 3% H_2_O_2_ for 30 min. Afterward, the slides were cleaned with water, and primary antibodies were incubated at 4°C for 12 h. Following washing, secondary antibodies were added for another 30-min incubation. Visualization was achieved using 3,3'-diaminobenzidine for color development (orange), with subsequent counterstaining with hematoxylin for nuclear staining (blue). The slides were then examined and photographed using a microscope.

### Statistical analysis

In this study, all data were analyzed using SPSS 22.0 software. The data were expressed as mean (SD). Among these, one-way analysis of variance (ANOVA) or two-way ANOVA was applied to compare differences between groups. Furthermore, we analyzed the difference between the two groups using the Student's t-test method. Statistical significance was defined as *P *<* *0.05.

## Results and discussion

### n-HA restrains the proliferation and invasiveness of glioma cells

**Figure 1.**
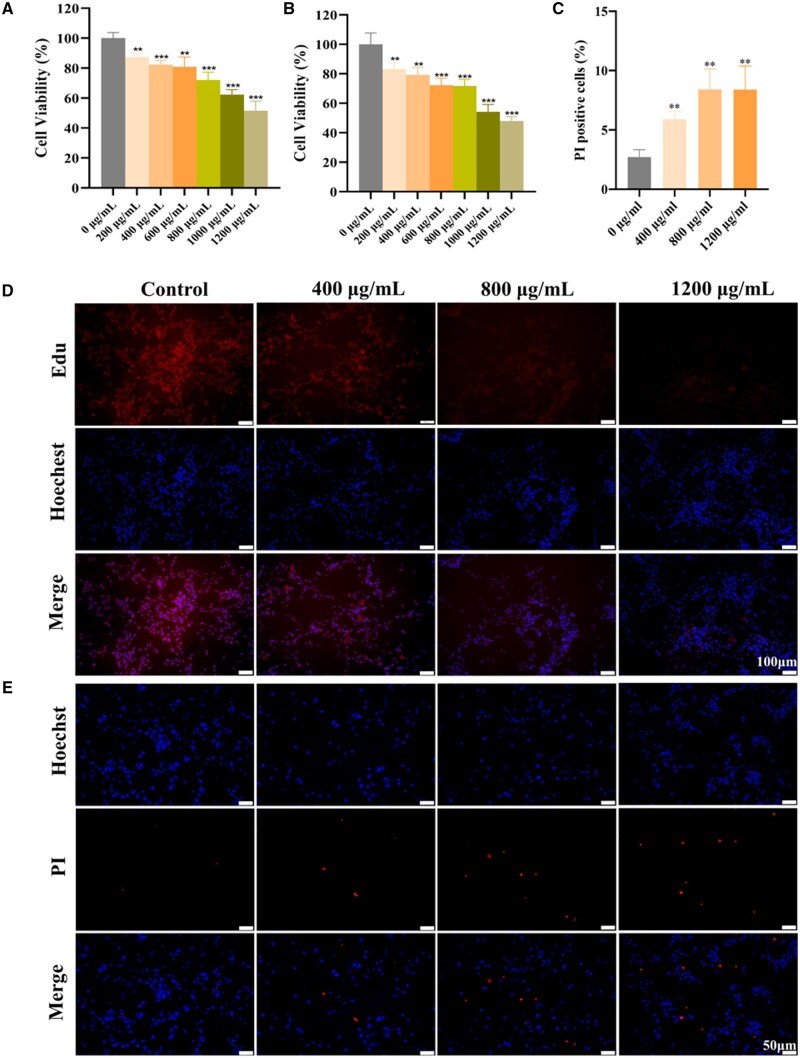
n-HA inhibited cancer cells’ proliferation and induced apoptosis in U87MG cells. (**A**, **B**) The viability of U87MG cells as detected via CCK-8 assay after 48 and 72 h treatment, respectively; (**C**, **E**) Representative images of Hoechst (blue)/PI (red) staining and semi-quantitative analysis of PI positive cells after treatment with various doses of n-HA for 48 h; (**D**) Representative images of Edu (red)/Hoechst (blue) staining. Scale bar = 100 μm (D) and 50 μm (**E**). ^**^P <* *0.01, ^***^P <* *0.001 vs Control.

According to the material characterizations, including TEM, XRD, FTIR and zeta potential tests (Supplementary Figures S1–S3 and Table S1), the n-HA exhibited a pure HA phase, needle-like morphology, nano-scaled particle size, and a negative zeta potential, which was consistent with the previous reports [25, 26, 29]. To investigate the influence of n-HA on glioma cells’ viability, various concentrations of n-HA were applied to U87MG cell line for either 48 or 72 h. Similar to earlier study [21], the vitality of U87MG glioma cells obviously declined at higher n-HA concentration (Figure 1A and B). When the n-HA concentration was increased to 1200 μg/ml, the cell viability rate dropped to nearly 50%, while the impact of increasing the cultivation time on cell viability was negligible. The Edu assays were then also carried out to determine the glioma cells’ proliferative activity, which was affected by n-HA (Figure 1D). The percentage of the Edu positive U87MG cells decreased as the increased n-HA concentration, with no discernible difference between the control group and those groups with n-HA concentrations of 400 and 800 μg/ml. A higher concentration of n-HA would result in the stopping of DNA replication and further reduction of cancer cell proliferation, as indicated by the significantly lower ratio of Edu positive cells in the 1200 μg/ml group compared to the control group (Supplementary Figure S4). Furthermore, the cell apoptosis after n-HA treatment was detected. Hoechst stains living cells in blue, and PI stains apoptotic cells in red. Compared to the control group, there was a significant increase in PI positive cells (Figure 1C and E). This indicated that n-HA caused U87MG cells to undergo apoptosis.

**Figure 2.**
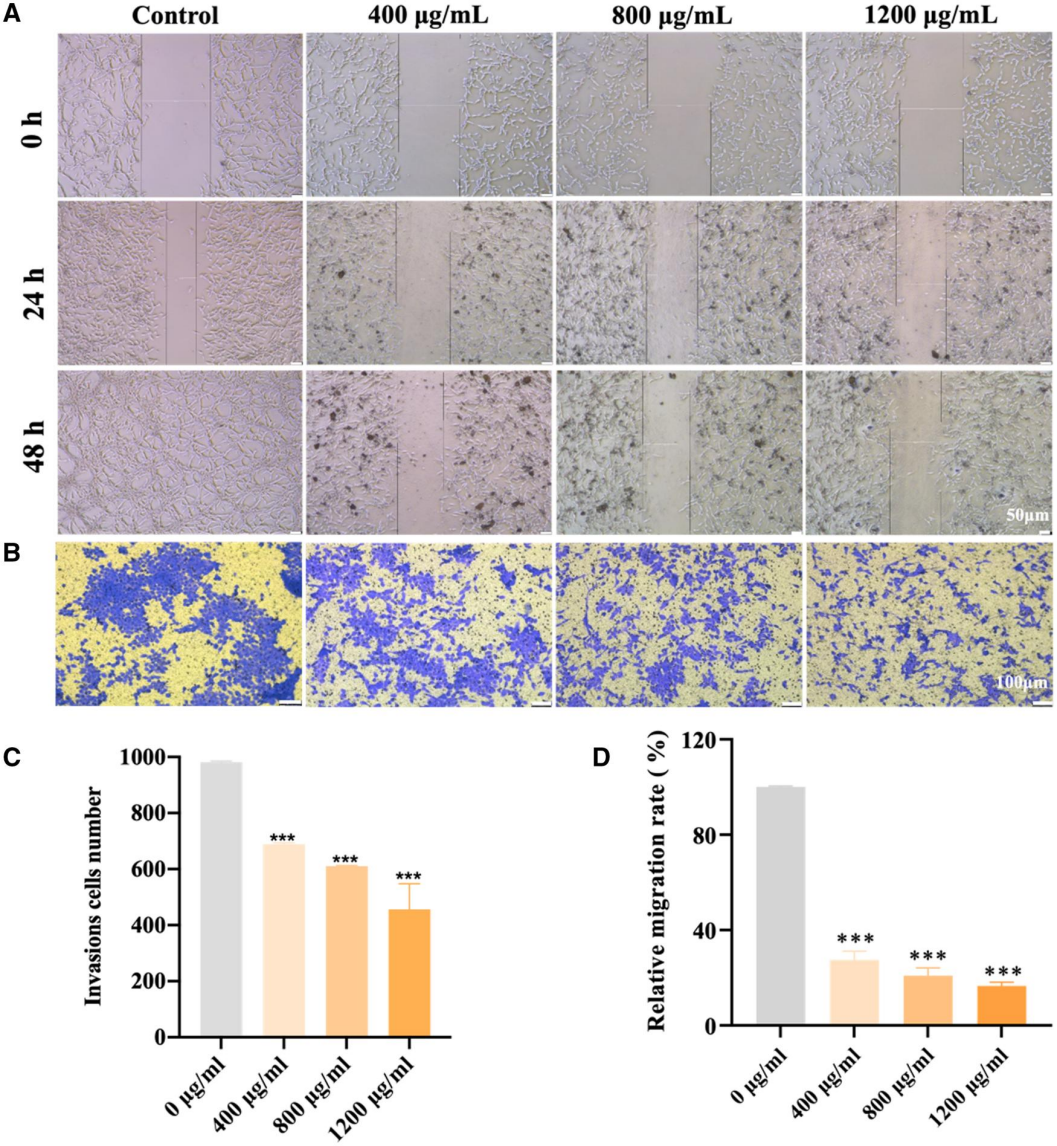
n-HA inhibited migration and invasion in U87MG cells. (**A**, **D**) Wound-healing assays showing migration level of U87MG cells after treatment with n-HA for 0, 24 and 48 h, respectively; (**B**, **C**) Transwell assays showing invasion level of U87MG cells after treatment with n-HA for 48 h. Scale bar = 50 μm (A) and 100 μm (B). ^***^P <* *0.001 vs Control.

In addition to cell growth, the metastasis of cancer cells is a significant contributor to patient mortality [43]. Therefore, we also explored the impacts of n-HA on cancer cell invasion and migration. As seen in Figure 2B and C, the number of blue-stained U87MG cells on the inversion transwell membrane decreased following treatment with n-HA, and this effect was significantly influenced with the incremental n-HA concentration, demonstrating that the invasion ability of U87MG cells suppressed by n-HA. Similar outcomes were indicated by the related wound-healing assays, where the rate of cancer cell migration slowed down following n-HA treatment (Figure 2A and D). As can be seen, the ability of n-HA, when its concentration increased to 1200 μg/ml, to inhibit tumor growth, invasion, and metastasis was the strongest, which was consistent with the results of inhibiting cancer cells’ proliferation. The previous study found that TR146 epithelial cell sheet displayed slower migration after n-HA treatment, which was ascribed to the increased cell contractility [44]. Therefore, the n-HA could disrupt the glioma cell migration by targeting the cytoskeleton.

### n-HA induces ER stress in glioma cells

**Figure 3.**
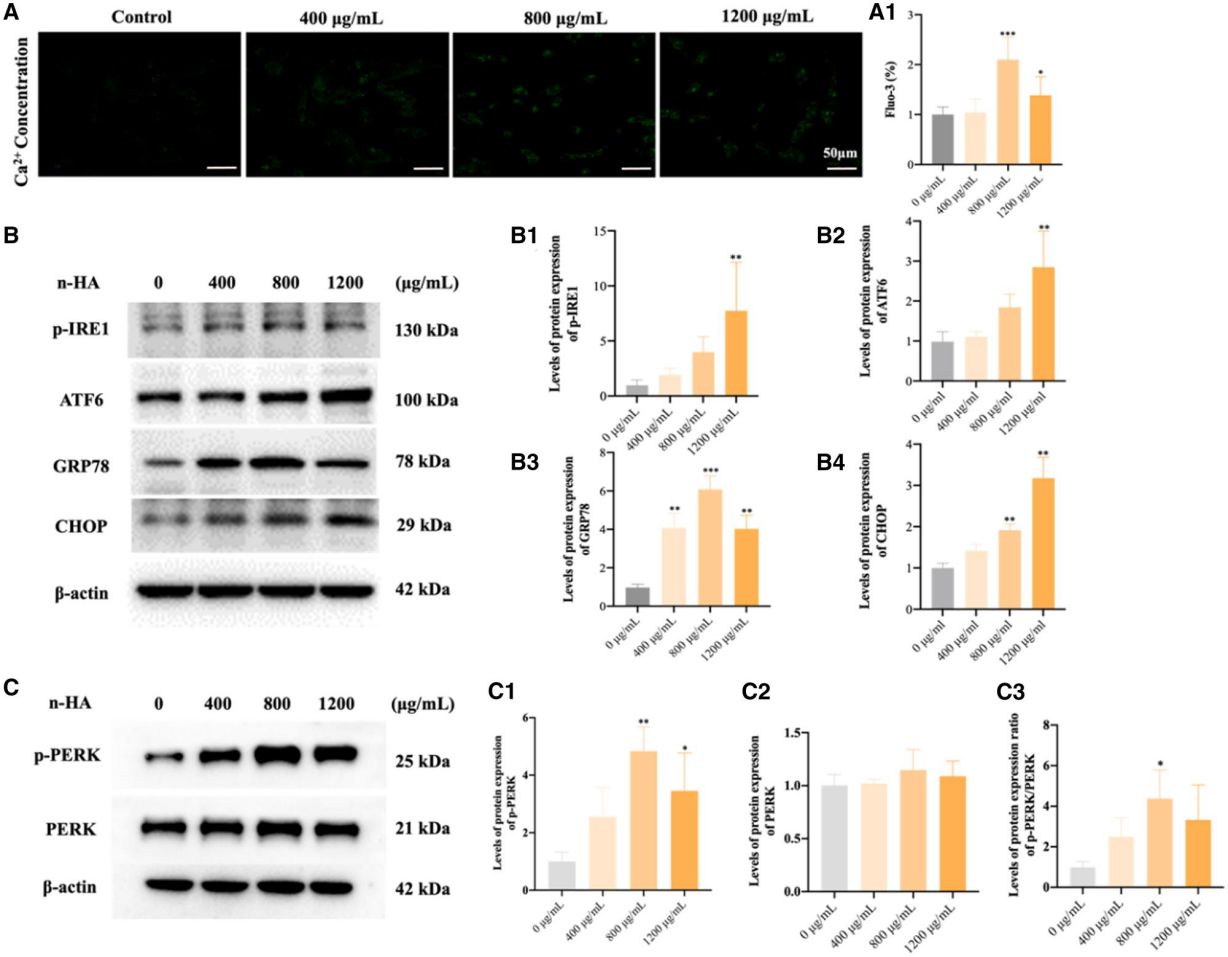
n-HA induced ER stress in U87MG cells. (**A**, **A1**) Intracellular Ca^2+^ ions concentration of U87MG cells after treatment with n-HA; (**B**, **C**) Western blot assay showing expression of ER-related proteins (p-IRE1, ATF6, GRP78, CHOP, p-PERK and PERK) after treatment with various doses of n-HA. Scale bars = 50 μm. ^*^P <* *0.05, ^**^P <* *0.01, ^***^P <* *0.001 vs Control.

Inspired by the effective anti-glioma properties of n-HA, we seek to unravel the mechanism using cellular and molecular knowledge. It is well acknowledged that n-HA would release Ca^2+^ during endocytosis, particularly in the acidic tumor microenvironment, which would cause Ca^2+^ to become overloaded and exacerbate cell death [45]. It is yet unknown, nevertheless, how excessive Ca^2+^ contribute to cell death exactly. In current study, the intracellular Ca^2+^ level indeed increased after n-HA incubated with U87MG cells, as demonstrated in Figure 3A (A1). As previously mentioned [46], Ca^2+^ is one of the most crucial second messengers, which participate in multiple cellular processes, like protein synthesis and secretion, cell cycle progression, and apoptosis. Since ER served as the primary intracellular Ca^2+^ store, its activities are significantly dependent on Ca^2+^ homeostasis. For instance, Ca^2+^-binding chaperones (e.g. calreticulin) are important for the correct folding and quality assurance during synthesis of new proteins in the ER. The unraveled protein response (UPR) and ER stress-induced apoptosis can also be triggered by an imbalance of ER Ca^2+^ [47, 48]. Hence, it is possible that n-HA contributed to the ER stress brought on by Ca^2+^ overload and further dysregulated cell activities as mentioned above.

First, the ER homeostasis was examined to confirm whether glioma cells were under ER stress. As shown in Figure 3B (B1–B4), glucose regulated protein 78 (GRP78) expression was upregulated after n-HA treatment compared to that in control group. GRP78, a molecular chaperone of the ER lumen, functions as part of the ER pathway. Cells depend on it for protein folding and assembly, as well as for cell regulation [49, 50]. In detailed, ER Ca^2+^ imbalance could cause the buildup of plenty of unfold proteins in ER, finally resulting in ER stress. To restore ER homeostasis, branches of the ER quality-control UPR system would be activated, including three branches activation, namely IRE1, PERK and ATF6 proteins [51]. As reported, these proteins’ luminal domains bind binding-immunoglobulin protein (BiP), which remains dormant in an unstressed state, which could be separated from BiP and been activated during ER stress [52, 53]. In this study, both p-IRE1 and ATF6 expression increased in n-HA groups. Moreover, compared with the control group, PERK was activated (phosphorylated forms), and p-PERK and PERK expressions were elevated after n-HA treatment as shown in Figure 3C (C1–C3). Taken together, all three UPR pathways (p-IRE1, PERK and ATF6) were activated, indicating the ER stress occurred under n-HA treatment.

Following that, further study was conducted on ER stress and apoptosis in glioma cells. As we know, CHOP is a marker gene for ER stress-induced apoptosis, and it can regulate this process [54]. Cells with declined CHOP gene expression significantly reduce ER stress-induced cell apoptosis. During ER stress, the UPR pathways are mainly responsible for inducing CHOP expression. For example, activated ATF6 stimulates the X-box binding protein 1 (XBP-1) and CHOP transcription under ER stress, and XBP-1 can further increase CHOP expression. Additionally, when IRE1α is activated under ER stress, it splices the XBP-1 mRNA introns to generate an active and mature XBP-1 protein, which increases the production of CHOP and further cell apoptosis [55–57]. Interestingly, as depicted in Figure 3B (B4), the expression of CHOP protein was observably increased after n-HA treatment, further confirming ER stress-induced apoptosis happened in glioma cells after n-HA treatment through the activation of CHOP. Moreover, the ability to upregulate p-IRE1, ATF6 and PERK expressions and the increased CHOP expression were all positively related with the n-HA concentration. These results agree exceptionally well with the inhibition of glioma growth, metastasis, and invasion. That indicates the activation of ER stress during n-HA treatment made a great contribution to the enhanced anti-tumor efficiency.

### n-HA induces mitochondria damage in glioma cells

As well-documented, mitochondria serve as significant but temporary Ca^2+^ buffers, and the proper Ca^2+^ concentration in mitochondria would activate vital metabolic processes like mitochondrial dehydrogenases [58, 59]. However, Ca^2+^ overload in mitochondria could be harmful to cells and even lead to cell death [60]. One potential explanation is that mPTP serves as a Ca^2+^-dependent channel, and the equalization of the proton gradient leads to mitochondrial depolarization. Subsequently, this depolarization results in the inhibition of cellular respiration and the excessive production of ROS. Additionally, mitochondria undergo swelling, leading to the release of intermembrane proteins [61].

**Figure 4.**
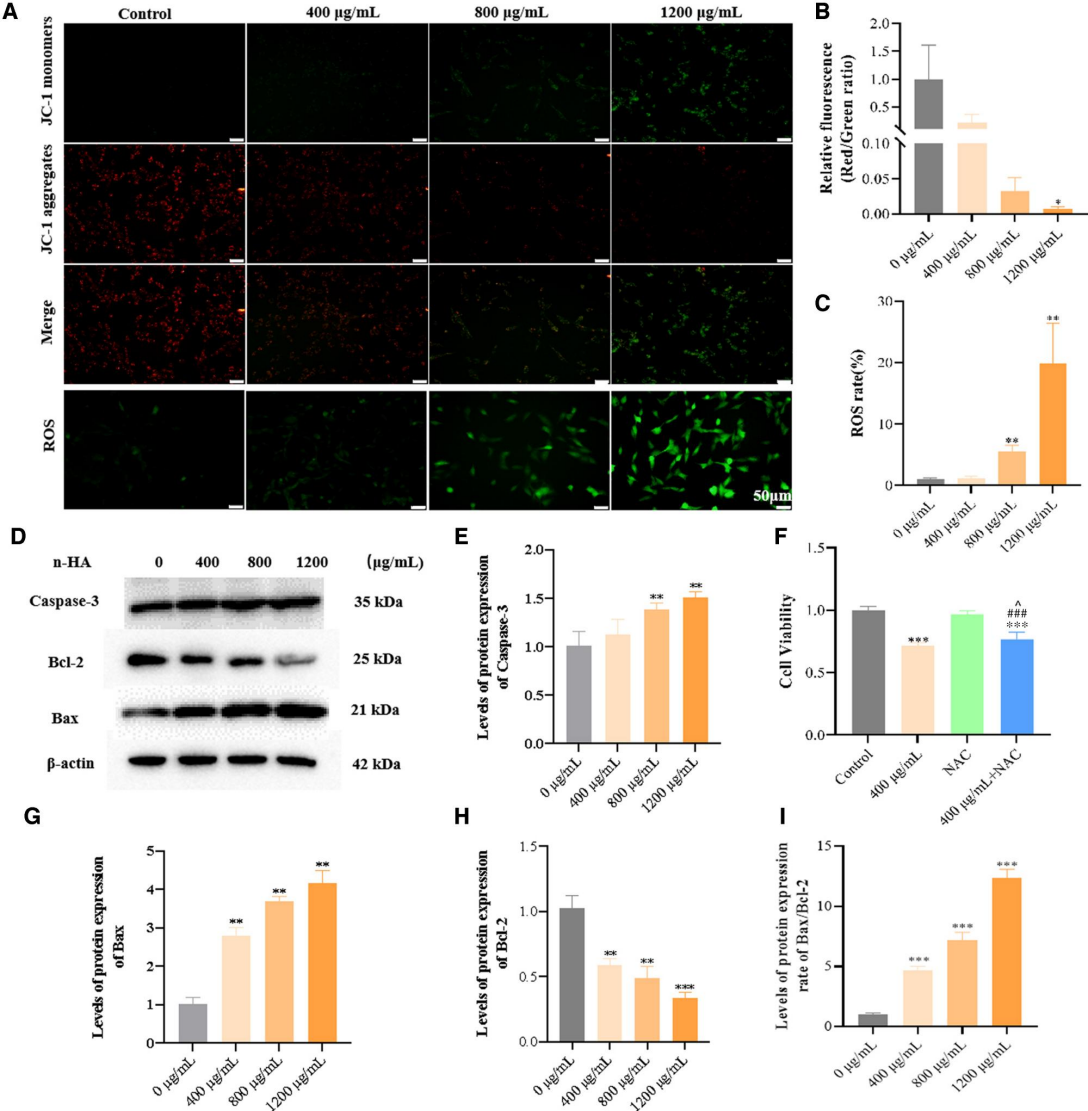
The impact of n-HA on mitochondrial damage in U87MG cells. (**A**, **B**) JC-1 staining and semi-quantitative analysis showing mitochondrial membrane potential of U87MG cells after treatment with various doses of n-HA; (A, **C**) Relative ROS rate and semi-quantitative analysis of U87MG cells after treatment with various doses of n-HA; (**D**, **E**, **G**, **H** and **I**) Western blot assay and semi-quantitative analysis showing expression of apoptosis-related proteins after treatment with various doses of n-HA; (**F**) Cells viability of U87MG cells after treatment with n-HA under different conditions tested by CCK-8 assay. Scale bar = 50 μm. ^*^P <* *0.05, ^**^P <* *0.01, ^***^P <* *0.001 vs Control. ^###^*P *<* *0.001 vs NAC group. ^^^*P *<* *0.05 vs n-HA-treated group.

To investigate the potential correlation between n-HA-induced apoptosis and mitochondrial damage, we initially used JC-1 to evaluate mitochondrial membrane potential. Under normal, non-apoptotic conditions, JC-1 dye forms aggregates within the mitochondrial matrix under normal, exhibiting an intense red fluorescence. Conversely, in aberrant or apoptotic cells where the membrane potential is lost, JC-1 cannot accumulate in mitochondria instead exists as a monomer in the cytoplasm, manifesting green fluorescence [62]. As illustrated in Figure 4A and B, the application of n-HA led to a progressive increase in green fluorescence within the cellular cytoplasm, indicating a dose-dependent disruption of mitochondrial membrane potential by n-HA, ultimately leading to its interference and deterioration. Meanwhile, the intracellular oxidation state was also examined using specific oxidation-sensitive fluorescent dye DCFH-DA. As depicted in Figure 4A, glioma cells treated with n-HA exhibited an increase of ROS with the increase of n-HA concentration. Using DCFH-DA staining as a fluorescent marker, the green fluorescence intensity in U87MG cells treated with n-HA for 48 h was significantly more pronounced compared to the control group, with the effect showing a dose-dependent trend (Figure 4C). We further explored the effect of ROS on n-HA inhibition of U87MG cell proliferation. As shown in Figure 4F, after treating U87MG cells with 400 μg/ml n-HA, NAC alone, and 400 μg/ml n-HA in combination with NAC for 48 h, cell activity was detected by CCK-8. It was found that cell viability increased after NAC treatment compared to the n-HA group alone, indicating that NAC reversed the n-HA-induced reduction in cellular viability. Hence, we can see that n-HA treatment indeed causes the mitochondrial membrane depolarization and ROS overproduction, further promoting cell death.

Furthermore, the WB results indicated that the n-HA treatment could result in the upregulated expression of cysteine-aspartic acid protease 3 (caspase-3) and pro-apoptotic Bcl-2 associated X protein (Bax), and downregulated B-cell lymphoma-2 (Bcl-2), indicating activation of the apoptosis signaling pathway after n-HA treatment. As reported, Bcl-2 proteins, which are mostly found on the outer membrane of mitochondria, are regarded as a key regulator of apoptosis [63], possibly being regulated by pro-apoptotic CHOP. Notably, the Ca^2+^ overload in mitochondrial could originate from the cytoplasm and ER. Even though the ER and mitochondria have different functions inside the cell, these organelles also physically connect with one another at locations known as mitochondria-associated ER membranes (MAMs), which permit Ca^2+^ transmit from the ER to the mitochondria under ER stress. It is anticipated that Ca^2+^ and ROS signals would interact locally, and that a self-amplifying loop involving calcium-induced ROS production and ROS-mediated calcium vulnerability will eventually trigger cell death in concert.

### n-HA induces glioma growth inhibition *in vivo*

**Figure 5.**
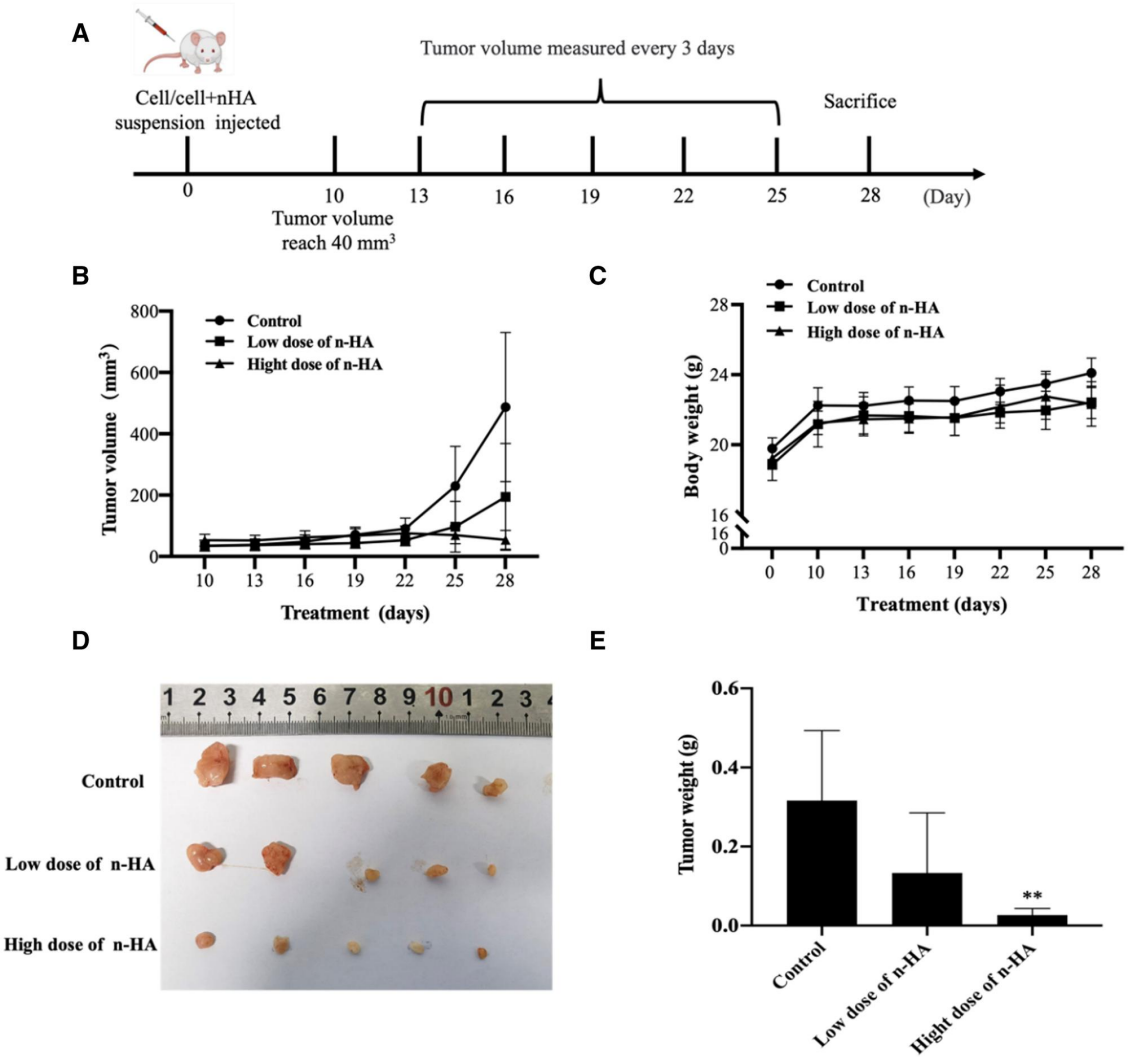
n-HA inhibits the growth of U87MG cells *in vivo* (*n* = 5). (**A**) Schematic illustration of the administration methods; (**B**) Tumor growth curves of subcutaneous models; (**C**) Body weight curves of mice in each group; (**D**) Images of the removed tumors in each group after 28 days; (**E**) Tumor weight of mice in each group after 28 days. ^**^P <* *0.01 vs Control.

We further investigated the roles and mechanisms of n-HA *in vivo* after being motivated by the positive anti-glioma impact of n-HA *in vitro*. The glioma animal model was constructed to verify whether n-HA could have the potential to inhibit tumor recurrence after glioma resection surgery. When used in local site, n-HA has been confirmed to have good biosafety [28, 29]. Our previous studies showed that the n-HA with the concentration of 200–400 µg/ml had excellent *in vitro* inhibiting effect on melanoma cell proliferation [25, 29]. However, in current study, a higher concentration of n-HA was required to inhibit U87MG glioma cell proliferation (Figure 1). Therefore, compared to our previous studies, higher doses of n-HA were used in current study, that is a low dose of n-HA (50 mg/kg) and a high dose of n-HA (100 mg/kg), were used to evaluate how n-HA affects the glioma growth *in vivo* (Figure 5A and Supplementary Figure S5). After subcutaneously injected into the back of the nude mice for 10 days, the tumor grew to ∼40 mm^3^ and was measured every 3 days in the following days. After 28 days, the tumor volume changes and weights were assessed after dissecting the tumor from the mice. As shown in Figure 5B, D and E, compared to untreated mice, n-HA-treated mice had smaller tumor sizes and weights, with the high dose of n-HA group showing a more significant. This indicated that n-HA can effectively inhibit the growth of U87MG cells. Additionally, the mice’s body weights also remained unchanged during treatment compared to the control groups (Figure 5C), demonstrating the biosafety of the n-HA used in glioma treatment.

**Figure 6.**
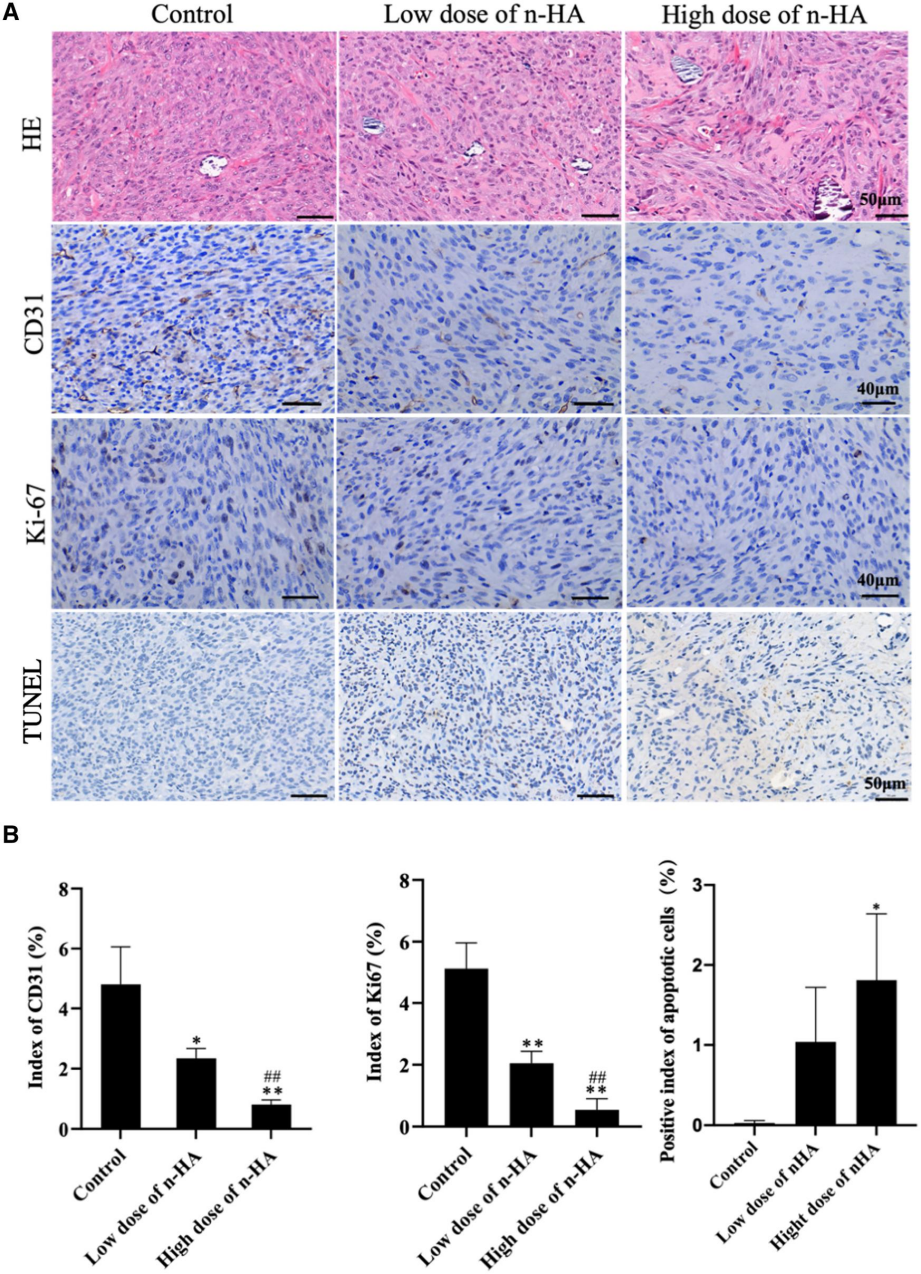
n-HA inhibits tumor cell proliferation and induces apoptosis *in vivo*. (**A**) Representative HE, CD31, Ki-67 and TUNEL staining images of tumor tissue; (**B**) Semi-quantitative analysis showing CD31 proteins expression, Ki-67 proteins expression and apoptotic cells of tumor tissues. Scale bar for HE and TUNEL staining = 50 μm. Scale bar for Ki-67 and CD31 staining = 40 μm. ^*^P <* *0.05, ^**^P <* *0.01 vs control. ^##^*P *<* *0.01 vs the low dose of n-HA group.

Subsequently, HE staining, IHC assay and Tunnel detection were used to test the hypothesis that n-HA *in vivo* reduced tumor cell proliferation and triggers apoptosis. Tumor tissue from each group was stained with HE, revealing that the nuclei in the n-HA-treated group had lighter color and looser cell organization (Figure 6A), indicating the n-HA’s potent anti-glioma activity. A tumor sample’s CD31 (a marker of endothelial cells) and Ki67 (a cell proliferation biomarker) levels were examined by IHC to further evaluate the *in vivo* anti-angiogenic and anti-proliferative effects of n-HA. According to previous studies [64, 65], tumor microenvironment would encourage endothelial cell infiltration via paracrine signaling pathways, which would lead to the creation of aberrant blood vessels and facilitate the spread of tumor cells. In this work, we discovered that the n-HA-treated tumors had lower Ki-67 and CD31 expression levels (Figure 6A and B). It is likely that n-HA might reduce the proliferative rate of glioma cells and trigger their apoptosis, which would alter the tumor’s microenvironment and reduce the infiltration of endothelial cells. Additionally, TUNEL staining results revealed that n-HA treatment dramatically accelerated glioma cell death in nude mice (Figure 6B).

**Figure 7.**
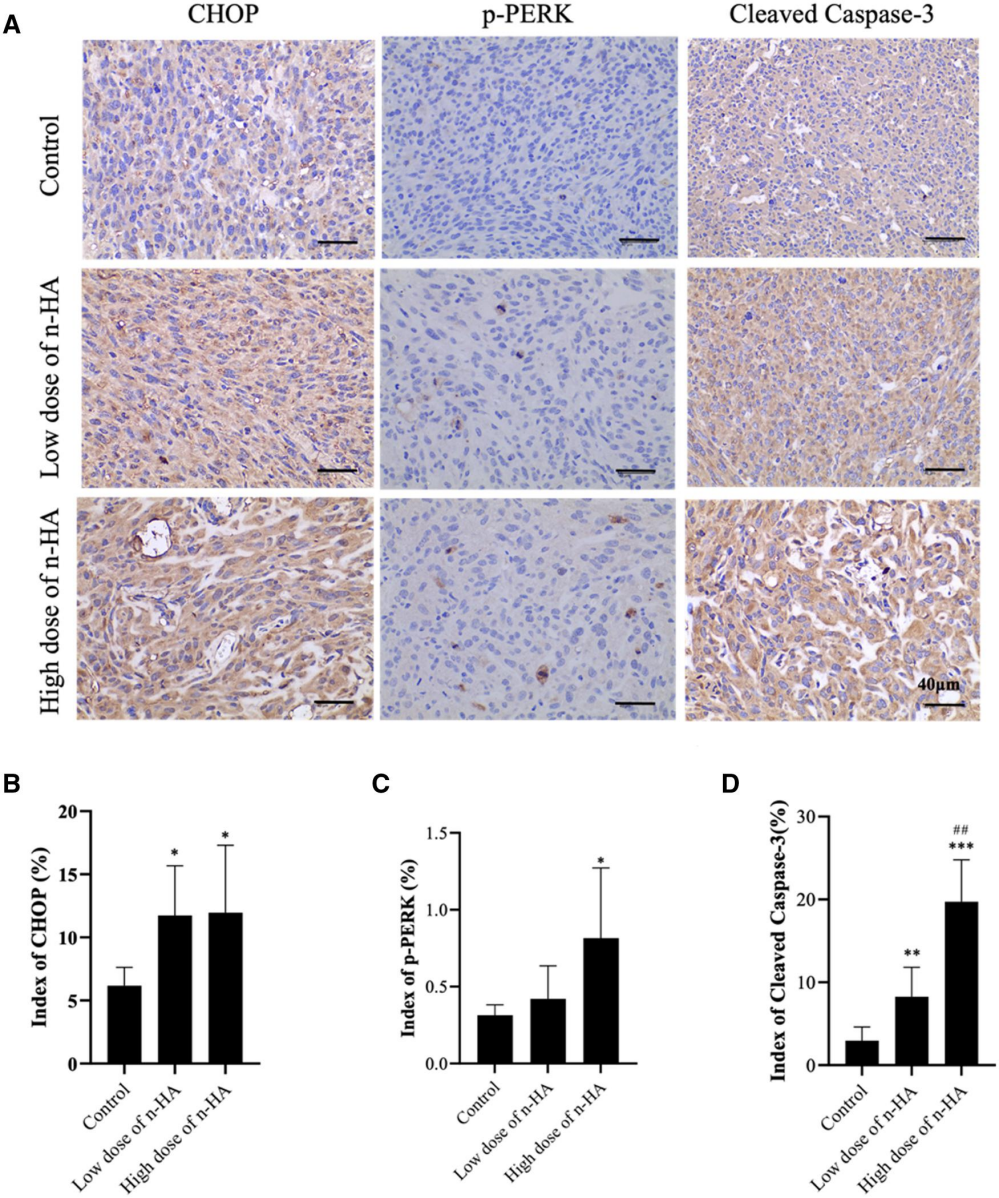
n-HA induced upregulated expression of ER stress and apoptosis related proteins *in vivo*. (**A**) Representative immunohistochemical staining images for CHOP, PERK and Cleaved Caspase-3 proteins in tumor tissues; (**B**–**D**) Semi-quantitative analysis showing CHOP, p-PERK and Cleaved Caspase-3 proteins expression in tumor tissues. Scale bar = 40 μm. ^*^P <* *0.05, ^**^P <* *0.01, ^***^P <* *0.001 vs Control. ^##^*P *<* *0.01 vs the low dose of n-HA group.

To further understand inherent mechanism causing the apoptosis of glioma cells, IHC analysis was employed in this study. As demonstrated in the cellular results, the expression of ER-related proteins (e.g. AFT6, p-PERK, etc,) was upregulated, triggering ER stress along with upregulating the expression of CHOP protein. CHOP is regarded as the predominantly pro-apoptotic transcription factor that triggers ER stress-mediated regulation of Bcl-2 family members, leading to apoptosis and further activation of the Caspase-3 apoptosis pathway. *In vivo* results further confirmed the findings from cell experiments. As shown in Figure 7, after n-HA treatment, the expression of ER-related proteins p-PERK and CHOP was upregulated, suggestive of ER stress induction. Figure 7A and D exhibited the highly expression of Cleaved Caspase-3 protein, and dose positive correlation, indicating that the high dose of n-HA treatment can induce glioma cell apoptosis and enhance the inhibition of glioma tumor growth. When all the information is considered, n-HA can effectively halt the growth of gliomas, which may be caused by the ER-stress and mitochondrial-damage induced death of gliomas. Hence, n-HA treatment could efficaciously reduce the growth of glioma tumors *in vivo* by inducing ER stress and mitochondrial damage, holding considerable promise.

## Conclusion

It is well known that n-HA has good anti-tumor activity, which is thought to be primarily associated with the Ca^2+^ overloading-induced cell death. However, ER and mitochondria, as the main Ca^2+^ storage and crucial modulators of cell apoptosis, have received little research regarding their roles in n-HA-induced cell apoptosis. In this study, we made needle-like n-HA to investigate their anti-glioma effectiveness and further elucidate the inherent mechanism. The proliferation and invasion of glioma cells were dramatically slowed down after n-HA treatment, according to data from *in vitro* tests. It is intriguing to observe that the expression of ER stress biomarkers, like p-IRE1, PERK, and ATF6, was upregulated, along with the activation of the transcription factor CHOP that promotes apoptosis, demonstrating the n-HA induced ER stress driven cell apoptosis happened. Meanwhile, the mitochondrial membrane depolarized and intracellular ROS, as well as pro-apoptotic genes were expressed at higher levels, demonstrating Ca^2+^ overload caused by n-HA could also promote cell apoptosis by co-inducing mitochondrial damage. The *in vivo* data gave more proof that n-HA successfully promote tumor cell apoptosis and halt glioma tumor growth. Overall, this work demonstrated that n-HA-induced intracellular ER stress and mitochondria damage could be crucial catalyst for cancer cells to undergo apoptosis. However, the current study has some limitations. Specifically, the research was confined to a single glioma cell line and a heterotopic tumor model. Going forward, it is imperative to explore the impact of n-HA on a broader range of glioma cell lines and to employ *in situ* glioma models, thus offering a more comprehensive understanding of its effects.

## Supplementary Material

rbae038_Supplementary_Data
