# Supplementary material for: Nano-hydroxyapatite promotes cell apoptosis by co-activating endoplasmic reticulum stress and mitochondria damage to inhibit glioma growth
Source: Regen Biomater. 2024 Apr 18;11:rbae038. doi: 10.1093/rb/rbae038 (PMC11127112; doi:10.1093/rb/rbae038)
Supplement: rbae038_Supplementary_Data [file rbae038_supplementary_data.docx]

**Supplementary Information**

**Nano-hydroxyapatite promotes cell apoptosis by co-activating endoplasmic reticulum stress and mitochondria damage to inhibit glioma growth**

Yifu Wang^1, 2^, Hongfeng Wu^1, 3^, Zhu Chen^1,4^, Jun Cao^1, 2^ (**🖂**), Xiangdong Zhu^1, 2^ (**🖂**), Xingdong Zhang^1,2^

^1^ National Engineering Research Center for Biomaterials, Sichuan University, Chengdu 610064, P. R. China

^2^ College of Biomedical Engineering, Sichuan University, Chengdu 610064, P. R. China

^3^ Medical School, Kunming University of Science and Technology, Kunming 650500, P. R. China

^4^ Institute of Tissue Engineering and Stem Cells, Nanchong Central Hospital, North Sichuan Medical College, Nanchong, 637000, P. R. China

Correspondence address. National Engineering Research Center for Biomaterials, College of Biomedical Engineering, Sichuan University, Chengdu 610064, P. R. China. Tel: +86-28-85412848; E-mail: [caojun@scu.edu.cn](mailto:caojun@scu.edu.cn) (Jun Cao), [zhu_xd1973@scu.edu.cn](mailto:zhu_xd1973@scu.edu.cn) (Xiangdong Zhu)


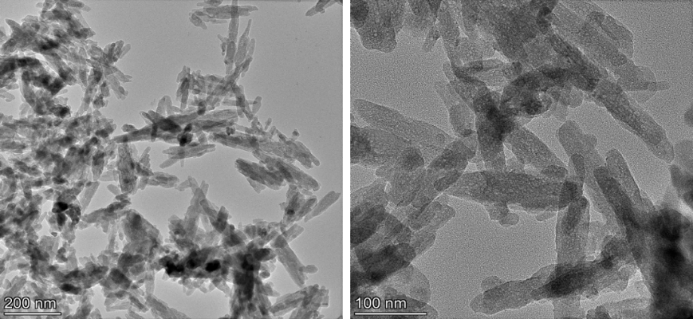


**Figure S1.** The typical TEM images of n-HA.


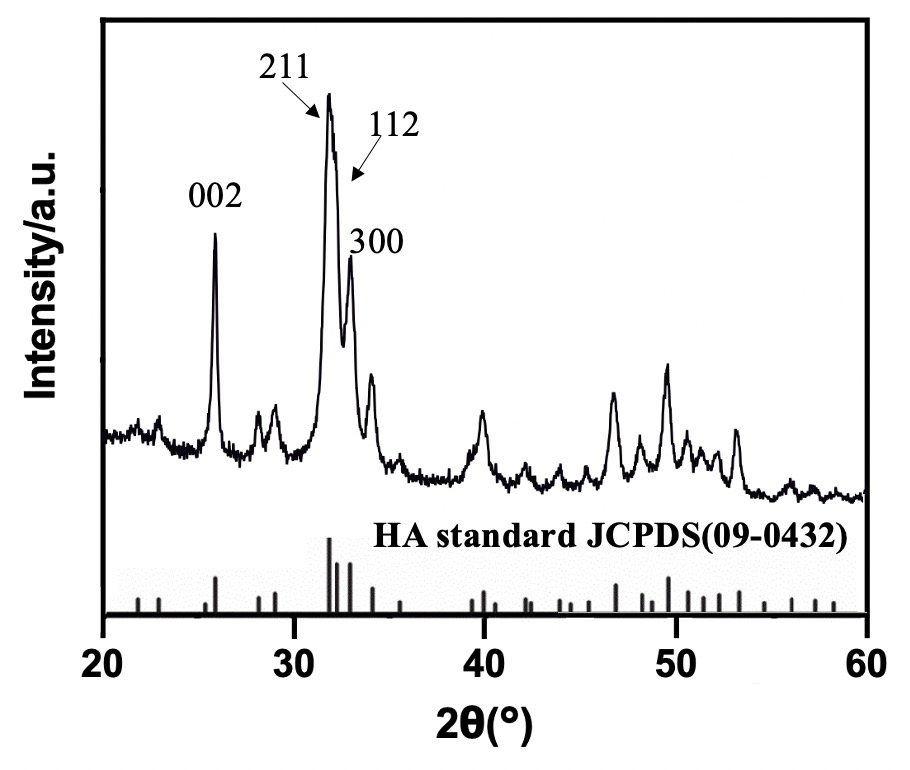


**Figure S2.** XRD pattern of n-HA.


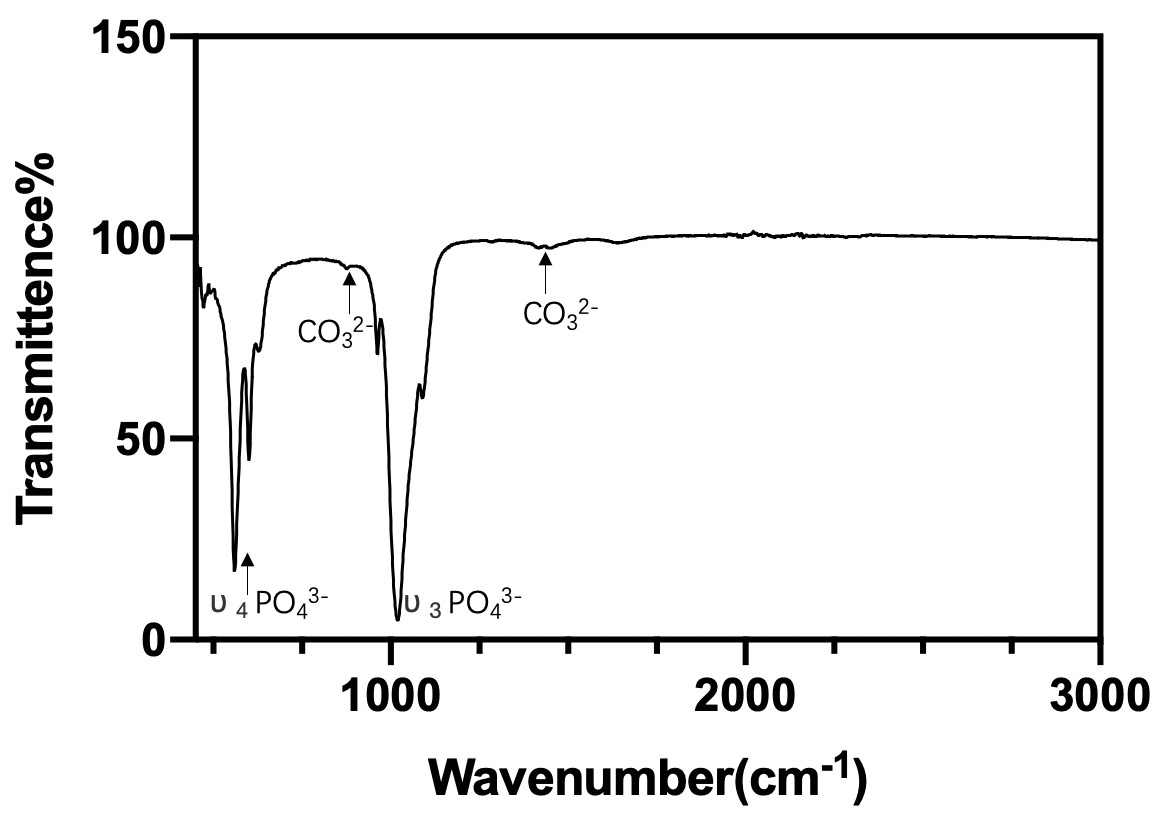


**Figure S3.**  FTIR spectrum of n-HA.


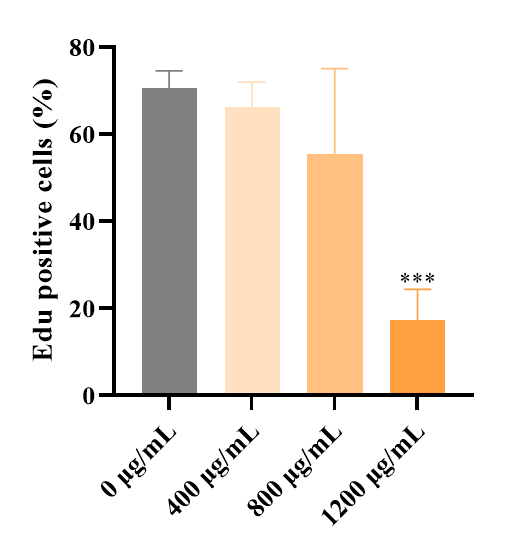


**Figure S4.** Semi-quantitative analysis of Edu positive cell. ^**^*p*<0.01, ^***^*p*<0.001 vs Control.


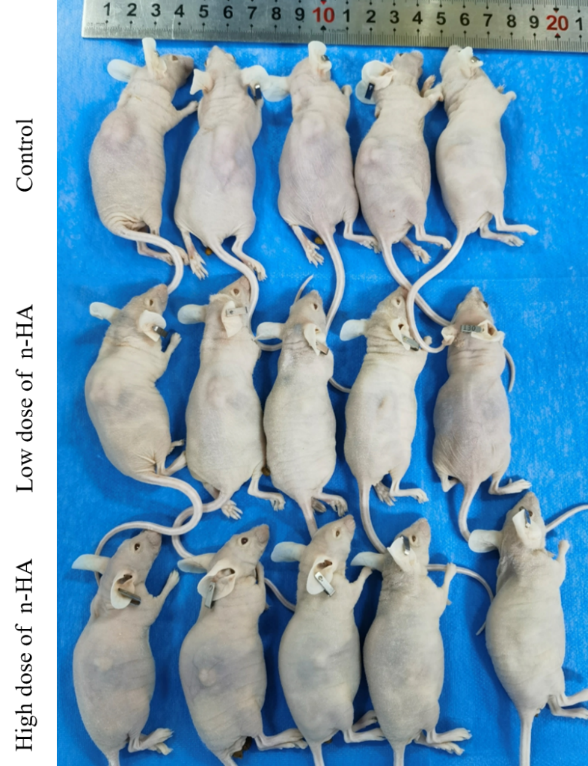


**Figure S5.** The gross observation for the glioma-bearing mice in each group after 28 days.

**Table S1.** Particle size and zeta potential of n-HA dispersed in DMEM solution

| Sample | Particle size (nm) | Zeta potential (mV) |
| --- | --- | --- |
| n-HA | 334.98±16.27 | -9.09±0.29 |
